# Vernix caseosa peritonitis – no longer rare or innocent: a case series

**DOI:** 10.1186/1752-1947-3-60

**Published:** 2009-02-10

**Authors:** Olivia A Stuart, Alastair R Morris, Rodney J Baber

**Affiliations:** 1 Department of Women's & Children's Health, Royal North Shore Hospital, Pacific Highway, St Leonards, NSW 2065, Australia; 2 Level 4, Wallace Freeborn Professorial Block, Royal North Shore Hospital, Pacific Highway, St Leonards, NSW 2065, Australia; 3 Medical Suites, 3rd Floor, North Shore Private Hospital, Westbourne St, St Leonards, NSW 2065, Australia

## Abstract

**Introduction:**

Vernix Caseosa peritonitis is a rare post caesarean section complication with only 19 case reports in the literature to date. Vernix caseosa spilt at the time of caesarean section is thought to incite an inflammatory reaction, causing symptoms resembling an acute abdomen.

**Case Presentation:**

We discuss three Caucasian patients (aged 32 to 43 years) who presented in our health sector in Sydney with vernix caseosa peritonitis. Each had a protracted course with significant comorbidities requiring surgical and medical intervention. This contrasts with other reports suggesting that a rapid resolution can be expected.

This cluster may be a consequence of the rising caesarean section rate, a heightened local awareness of the condition and possibly a result of leaving material in the paracolic gutters intraoperatively.

**Conclusion:**

Our aim is to increase awareness among our obstetric and surgical colleagues of the characteristic clinical presentation and intra-operative findings of vernix caseosa peritonitis. We also point out that, in contrast to those presented here, not all patients require laparotomy.

## Introduction

There have been only 19 cases of vernix caseosa peritonitis (VCP) reported in the literature to date [1-13]. We present three cases that have occurred within a 24-month-period in our regional health area in Sydney, now comprising 14% of the total reported cases.

The typical presentation is severe abdominal pain [1-12,14], pyrexia [1-3,5-12,14] and peritonism [1-3,6,7,9,10,14] within 3 to 35 days following caesarean section (CS) [14] with the majority representing after discharge. There is a neutrophilic leucocytosis on full blood count and vaginal, urinary and haematological microbiological specimens are negative. Investigations to exclude more common causes of postoperative peritonitis such as plain abdominal X-ray, erect chest X-ray and ultrasound, computed tomography (CT) and magnetic resonance imaging (MRI) of the abdomen and pelvis show non-specific changes or are normal. Diagnostic laparoscopy or laparotomy is performed to exclude the other more common pathologies (i.e. appendicitis, bowel/ureteric injury, endometritis or a ruptured viscus). The laparoscopic view has a characteristic appearance with cheese-like plaques of vernix caseosa (VC) deposited on visceral and parietal peritoneal surfaces throughout the abdominal cavity. Peritoneal or omental biopsies are diagnostic, showing an acute, chronic, or mixed inflammatory infiltrate centred on anucleate fetal squamous cells, depending on the time from exposure to diagnosis.

Previously, many additional and often extensive excisional procedures have been performed on suspicion of other pathologies such as appendicectomy [1,3,5,7,9], cholecystectomy [11], total abdominal hysterectomy [2,5], salpingo-oophorectomy [2,5,9] and colectomy [4,6,8] with normal subsequent histology. Authors remark on a hasty and complete resolution of symptoms following conservative management of analgesics, antibiotics and sometimes steroids in resistant cases where infective aetiologies have been excluded [9] with no cases reporting problems more than 2 weeks following delivery. Our three cases have experienced more protracted problematic courses and we discuss these further. We aim to highlight this complication and believe that VCP may be more common and not as innocent as was previously thought.

## Case presentations

### Case one

A 40-year-old nulliparous Caucasian woman was delivered by an uncomplicated elective lower segment caesarean section (LSCS) at 39 weeks gestation for breech presentation after an unremarkable antenatal course. She developed generalised peritonism, dyspnoea and pyrexia (39.3°C) 5 days later. A neutrophilic leucocytosis (white cell count (WCC) 15 × 10^9^/L) was present but both an abdominopelvic CT and CT pulmonary angiogram (CTPA) were normal. At laparotomy, cheese-like fibrinous debris was found throughout the peritoneal cavity. Peritoneal lavage was performed and an omental biopsy obtained after exclusion of other pathologies and on suspicion of VCP. Histological examination showed acute fibrinous inflammation of the serosal surface and early epithelioid granulomata associated with fetal squamous cells. Antibiotics were commenced and her symptoms rapidly resolved allowing discharge 5 days post-laparotomy. Five weeks later, she was readmitted to hospital for management of recurrent abdominal pain. All her blood tests and an upper abdominal ultrasound were normal. She was managed conservatively and discharged 3 days later. Ten weeks post-delivery, she represented for a second time, now with a persistent discharge from the edge of the abdominal incision. An abdominal wall wound sinus originating superficial to the rectus sheath was identified and excised allowing a rapid resolution of her symptoms. A collection of fetal squamous cells and a granuloma containing VC was observed within the sinus tract that was lined with inflammatory granulation tissue containing aggregates of histiocytes and multi-nucleated giant cells. Two years later, she has continued to have ongoing pain culminating in a recent laparoscopic excision of a VC abscess on the anterior abdominal wall adjacent to the liver, confirmed on histopathology.

### Case two

A 32-year-old primiparous Caucasian woman had previously had a normal vaginal delivery but underwent elective LSCS at 39 weeks gestation for breech presentation. She had had a normal medical and antenatal history and although the LSCS was uncomplicated, she developed abdominal pain, flu-like symptoms and myalgias 3 days post-delivery. A diagnosis of urinary tract infection was made on the sixth postoperative day as she now complained of dysuria and her temperature was 41°C. Oral antibiotics were commenced and her condition improved, allowing her to be discharged. On day eight, she represented with further pyrexia, dysuria and severe abdominal pain requiring opiate analgesia. Urine culture was negative and an abdominopelvic ultrasound scan was normal. Once more, her symptoms settled with intravenous antibiotics and she was discharged a week later. She continued to experience intermittent episodes of pain culminating in a third admission 3 months postpartum. On this occasion, abdominopelvic CT and MRI identified an 8 cm inflammatory mass involving the wound. Laparotomy revealed a wound abscess and sinus formation tracking to the broad ligament that required extensive debridement and division of adhesions. Biopsies from the wound and inflammatory mass showed a chronic inflammatory granulomatous process associated with extravasated fetal squames and foreign material. A month later, she experienced further pain and dysuria. She underwent extensive investigation for thrombo-embolism, osteomyelitis, tuberculosis and further histological and microbiological investigation of wound biopsies that were normal. A repeat histopathological review of her previous biopsies diagnosed VCP. A urological opinion concluded that paravesicular inflammation was responsible for her urinary symptoms. In all, her pain continued for 7 months post-caesarean section before resolving.

### Case three

**Figure 1:**
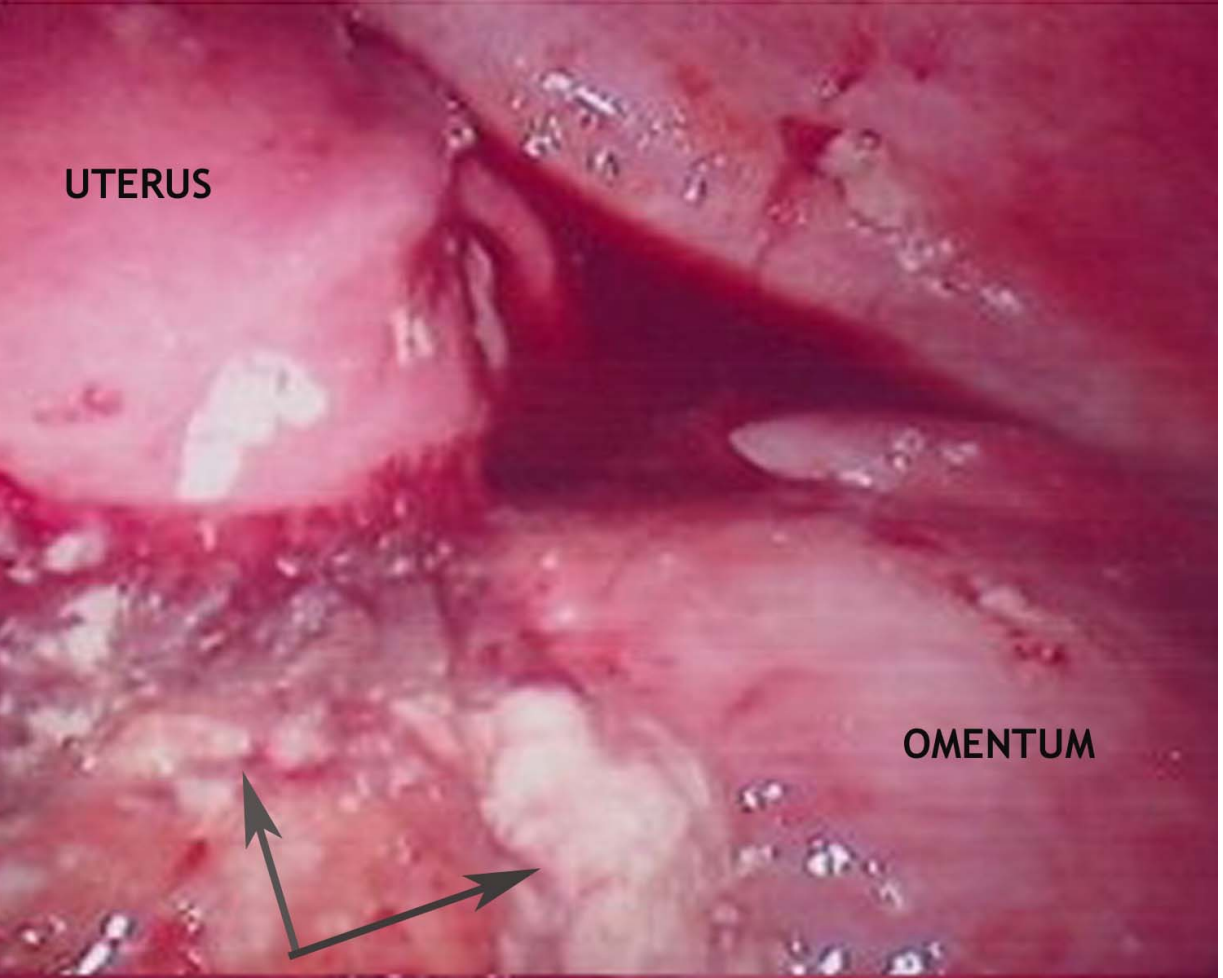
**Laparoscopic view of vernix caseosa peritonitis**. Vernix caseosa deposits are scattered throughout the peritoneal cavity (grey arrows) in this laparoscopic view of the pelvis; Case 3.

**Figure 2:**
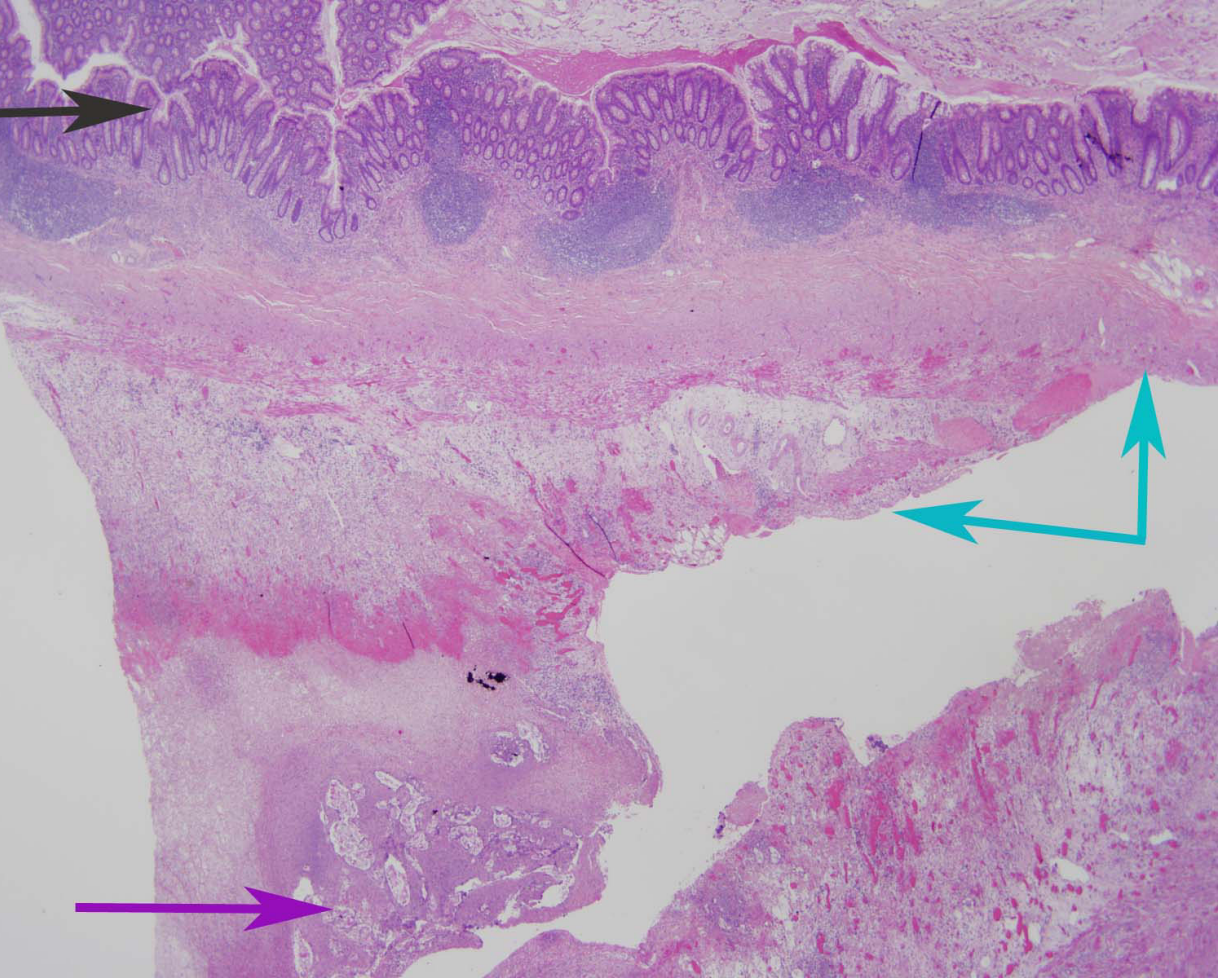
**Low power view of an appendiceal biopsy showing vernix caseosa peritonitis**. This low power view of the appendiceal biopsy shows normal mucosa and underlying muscularis propria (black arrow), and a serosal inflammatory infiltrate (blue arrows) centred around fetal squamous cells (purple arrow); Case 3 (haematoxylin and eosin staining, 20× magnification).

**Figure 3:**
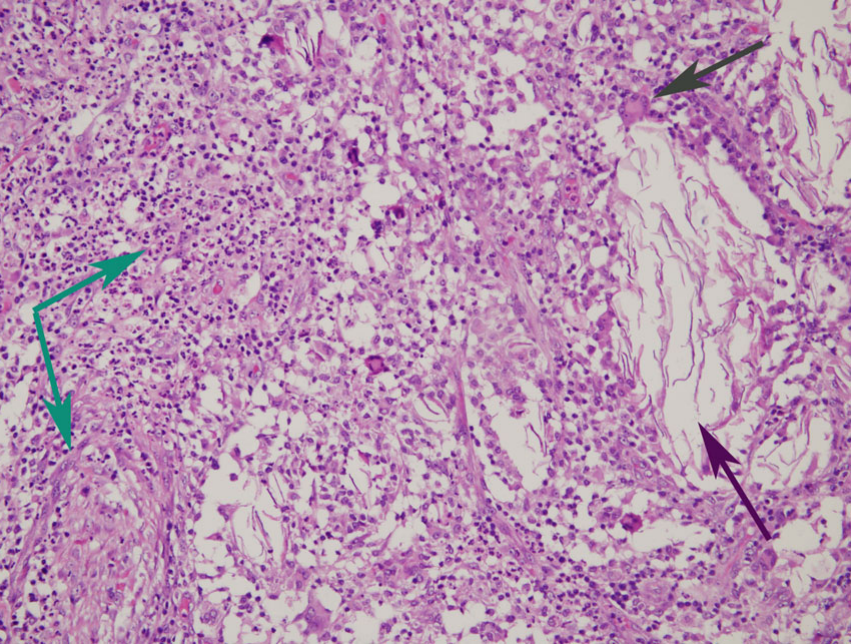
**High power view of vernix caseosa peritonitis**. This high power view shows a mixed inflammatory infiltrate, comprising neutrophils, histiocytes (green arrows) and giant cells (black arrow) centred on aggregates of anucleate fetal squamous cells (purple arrow); Case 3 (hematoxylin and eosin staining, 400× magnification).

A 43-year-old woman had had 12 first trimester miscarriages and terminations of pregnancy before the current pregnancy with an otherwise unremarkable medical and antenatal history. She underwent an uncomplicated emergency LSCS for failure to progress in the first stage of labour. Four days post-delivery, she developed shoulder-tip pain in association with abdominal distension, peritonism and pyrexia. An abdominopelvic CT was unremarkable and as her clinical picture was not improving despite analgesia and antibiotics, a laparoscopic investigation was undertaken. VC extruded through the Hassan port at entry and was also scattered throughout the peritoneal cavity (Figure 1, grey arrows). The procedure was converted to laparotomy through the Pfannenstiel incision, and adhesiolysis, lavage and peritoneal biopsy performed. Histology revealed an acute inflammatory exudate forming in response to fetal squames and keratin debris. Her recovery was slow and complicated by persisting pain and abdominal distension. By day 10, the abdominal distension had still not adequately settled despite nasogastric aspiration and parenteral nutrition. Consequently, a contrast CT was undertaken which showed a high-grade small bowel obstruction. A second laparotomy was performed – this time through a midline incision. The cause of the obstruction was identified as a large inflammatory mass of adhesions involving loops of small bowel. Adhesiolysis, omentectomy, appendicectomy and abdominal lavage were performed. Her appendiceal biopsy showed normal mucosa and underlying muscularis propria (Figure 2, black arrow), and a serosal inflammatory infiltrate (Figure 2, blue arrows) centred around fetal squamous cells (Figure 2, purple arrow). Interestingly, the inflammatory infiltrate was now mixed, comprising neutrophils, histiocytes (Figure 3, green arrows) and giant cells (Figure 3, black arrow) centred on aggregates of fetal squamous cells (Figure 3, purple arrow). Her symptoms rapidly resolved allowing her discharge home but she had subsequent admissions over the following 6-month period with subacute small bowel obstruction. On each of these occasions, she responded to conservative treatment and as yet, no further surgical interventions have been required.

## Discussion

We have presented three cases of post-caesarean section peritonitis in patients from the same health sector over a 24-month period. The two adjacent maternity units involved have 2600 and 2400 deliveries per year with caesarean section rates of 42% and 33%, respectively. Both rates have increased 2.5% every 5 years over the last 20 years that we have records for and both surpass the national average rate of approximately 30%, leading us to believe that an increasing CS rate, with a consequent increase in caesarean complications, is responsible for this cluster. Certainly, the incidence in our area of late has heightened awareness of this entity among our local obstetric, surgical and histopathological community and with increasing awareness in the general community, we may see a paradoxical rise in reported cases that may have previously been undiagnosed or assumed to be due to other disease processes. Mopping the paracolic gutters of excess debris and blood before closure to reduce postoperative pain has been traditional teaching and is certainly routine practice within our teaching hospital and those surgeons involved in all our cases. That said, the increasing caesarean section rate we are seeing, mirroring that of the nation, may contribute to more surgical laxity in this area and an increased incidence of VCP.

The health population in our area comprises a moderate to high socio-economic group and ages of our patients ranged from 32 to 43 years of age where there may be an as yet undiagnosed element of immunological hypersensitivity. If this is the case, then the volume of VC needed to incite a reaction is probably small and mopping of the paracolic gutters may not be helpful. However, two of our patients were primipara suggesting that hypersensitivity reaction is less likely given there has been inadequate time for sensitisation to occur, even from an antenatal priming event. In keeping with hypersensitivity reactions, there are too few cases reported to see whether multipara have a more exaggerated response and this is a potential focus for future research.

The principal symptoms of VCP are generalised severe abdominal pain, pyrexia, peritonism and elevated white cell count with inconclusive or normal imaging. Other causes of peritonism are more likely including intraperitoneal sepsis, endometritis and iatrogenic ureteric/bowel injury. We agree that there should be no hesitation in undertaking further emergency laparoscopic or open surgical investigation should the clinical presentation be such that it warrants exclusion of these other more common pathologies. However, the observation of white cheese-like plaques and/or VC within the peritoneal cavity upon entering should raise the suspicion of VCP. Appropriate serosal biopsies are needed to confirm the diagnosis in the absence of other identifiable aetiologies. Our review of the literature found that most reported cases had significant additional procedures of laparotomy that, with hindsight, may have compromised recovery. The subsequent normal histological findings in the excised organs highlight the need for improved awareness among surgeons to reduce the morbidity from additional surgery.

Various conservative treatments have been tried although many authors omitted to outline their postoperative management [2,4-7,13]. Some advocated postoperative antibiotic therapy [1-3,8-12]. Adjuvant steroid therapy was used in two cases with resistant symptoms where infection had been excluded [9]. Facilitation of recovery was achieved in both of these cases and the authors postulated that steroids had significantly enhanced the clinical course by suppression of the inflammatory response. However, all of our cases developed significant morbidities following the initial diagnosis of VCP that did require further operative procedures. Complications such as bowel obstruction are life-threatening showing that this condition is not entirely benign. Our review of the literature suggests that VCP is generally a self-limiting condition and resolves with conservative management alone. However, our experience suggests that this is not always the case and monitoring the postoperative course of those diagnosed with VCP is important as delayed morbidities may arise.

## Conclusion

With an increasing caesarean section rate, the incidence of post-caesarean complications such as vernix caseosa peritonitis is also rising. Obstetricians should be aware of this condition to avoid unnecessary invasive procedures with the mainstay of management being exclusion of other more common pathologies, obtainment of biopsies to achieve histological diagnosis, and analgesia. The pathological process seems not as innocuous as once was thought and vigilant monitoring after diagnosis is required as delayed morbidities may arise necessitating timely intervention.

## Consent

Written informed consent was obtained from the patients for publication of this case series and any accompanying images. A copy of the written consent is available for review by the Editor-in-Chief of this journal.

## Competing interests

The authors declare that they have no competing interests.

## Authors' contributions

RB conceived writing this case series after the initial patient presented with VCP in his practice and the second case presented in a colleague's practice. OS performed the literature search, compiled the case histories and results, and wrote the skeleton manuscript. AM and OS were both involved in the care of case three. All authors read, edited and approved the final manuscript.
